# Disturbance Observer-Based Model Predictive Control for Multi-Frequency Interference Suppression in Space Laser Communication Systems

**DOI:** 10.3390/s26103182

**Published:** 2026-05-18

**Authors:** Yang Chen, Mochi Tan, Lin Ge, Yaoyuan Zhang, Feng Qin, Long Chen, Siyuan Yu

**Affiliations:** 1 School of Instrumentation Science and Engineering, Harbin Institute of Technology, Harbin 150001, China; 2 School of Computing Science, Simon Fraser University, Burnaby, BC V5A 1S6, Canada

**Keywords:** laser communication, beam pointing control, predictive control

## Abstract

High-precision beam pointing control is essential for achieving reliable data transmission in space laser communication systems, requiring effective suppression of multi-frequency disturbance-induced tracking residuals. To address this issue, a synergistic model predictive control and disturbance observer (DOB-MPC) architecture is developed. The proposed hierarchical control framework is constructed based on gimbal servo dynamics and implemented through the following steps. A frequency-decoupled disturbance observer (DOB) is employed to estimate multi-frequency disturbances using spectral separation principles, while a model predictive controller (MPC) enhances steady-state accuracy by compensating for time-delay effects through trajectory optimization. Crucially, the disturbance estimates are incorporated into the MPC’s prediction horizon via receding-horizon optimization, establishing a cooperative control mechanism. Simulation results demonstrate improvements of greater than 25% in dynamic response and over 80% in residual error reduction compared to conventional methods. Experimental results further validate the efficacy of the proposed architecture for space laser tracking systems.

## 1. Introduction

Conventional radio frequency wireless communication technologies suffer from inherent drawbacks in free space scenarios, including high power consumption, low data rates, and restricted effective operating ranges [1]. Space laser communication utilizes optical carriers for data transmission, enabling long communication distances and high data rates owing to its narrow beam divergence angle. This advantage makes it highly attractive in response to the rapidly growing demand for high-speed communication [2,3,4].

Given the ultra-narrow divergence of laser beams, the accuracy of beam steering should be maintained on the order of micro-radians (μrad) to ensure comprehensive coverage of links in different scenarios, including aerospace, ground, and underwater environments. Therefore, a high-precision control system is required to maintain stable beam alignment under dynamic disturbances. Consequently, the development of high-precision pointing, acquisition, and tracking (PAT) technology plays an important role in establishing robust space laser communication links [5].

PAT systems for space laser communication typically adopt a dual-loop control architecture consisting of coarse and fine tracking subsystems [6,7,8]. The coarse tracking loop is responsible for rejecting large-amplitude disturbances and maintaining the beam within the acquisition range, while the fine tracking loop further refines the pointing accuracy. To ensure proper operation of the fine tracker, the residual error of the coarse loop must be constrained within a fraction of its field of view (typically 0.01–0.005°), imposing stringent requirements on coarse tracking performance [9].

Such high-precision pointing is critical because residual errors directly affect the coupling efficiency of the received optical signal into the detector or single-mode fiber. Improved coupling efficiency enhances the optical signal-to-noise ratio (SNR) and reduces the bit error rate (BER), thereby ensuring reliable communication links. Therefore, achieving both high dynamic response and precise residual error suppression in the coarse tracking loop is essential for dual-loop PAT systems [10,11].

A variety of advanced control methods have been developed to enhance coarse tracking performance. Traditional Smith predictor-based control compensates for fixed time delays but shows limited adaptability to time-varying delays and disturbances, and high-gain filter design may compromise stability [12,13]. Active Disturbance Rejection Control (ADRC) improves disturbance rejection through extended state observation, yet its performance in high-dynamic scenarios is constrained by the trade-off between observer bandwidth and noise sensitivity [14]. Similarly, Linear Quadratic Regulator (LQR) methods enable rapid response but are less effective under model uncertainties and coupled multi-frequency disturbances, leading to insufficient disturbance rejection [15].

Meanwhile, extensive research has been dedicated to advanced control architectures for residual error attenuation. The Proportional-Integral-Derivative with Disturbance Observer (PID-DOB) composite architecture suppresses steady-state errors through integral action. However, its linear compensation mechanism is inadequate to suppress time-varying disturbances in the 0.1–1 Hz band. Moreover, saturation of the control input may lead to a deterioration of the phase margin [16]. Model Predictive Control (MPC) implicitly compensates for time delays through receding-horizon optimization and future dynamics prediction using system models, demonstrating robust delay compensation and parameter perturbation tolerance. However, its performance can be susceptible to modeling inaccuracies and coupled multi-frequency disturbance effects, often leading to insufficient suppression of residual errors [17,18,19,20].

Therefore, it is challenging to simultaneously realize high dynamic response and accurate error suppression in coarse tracking, which necessitates effectively addressing both interference suppression and time-delay compensation in dual-loop PAT architectures. The primary disturbance source originates from platform perturbations, characterized by low-frequency (0.1–1 Hz), high-amplitude, and time-varying dynamics [21,22,23]. Concurrently, detection and transmission processes introduce millisecond-level delays. Therefore, maintaining sub-milliradian tracking stability and robust link performance under dynamic operational conditions critically depends on the accurate compensation of both external disturbances and inherent system delays. These factors lead to spectral overlap between disturbances and measurement noise, significant phase lag, and model uncertainty, which in turn cause bandwidth limitations and competition in the frequency domain, ultimately degrading system stability [24].

Disturbance-observer-based MPC (DOB-MPC) has emerged as an efficient approach to enhance disturbance rejection and tracking accuracy. In most existing approaches, disturbances are typically modeled as input-side uncertainties and incorporated into the prediction model as augmented states or feedforward compensation terms. While such formulations are effective for certain classes of disturbances, they may fail to accurately capture the characteristics of output-dominant disturbances, such as optical-axis perturbations in space laser communication systems.

In disturbance-estimation-based MPC methods, disturbance estimates are often treated as constant preview signals over the prediction horizon. When incorporated in this manner, they remain independent of the control sequence and thus have limited influence on the optimization process, restricting the effectiveness of disturbance-aware control.

Moreover, existing DOB-MPC designs rarely provide explicit coordination between disturbance estimation and predictive control in the frequency domain. This can lead to bandwidth competition between the disturbance observer and the controller, resulting in degraded disturbance rejection performance, particularly in the presence of multi-band disturbances and time-delay effects.

These issues motivate the development of a DOB-MPC framework that explicitly accounts for output disturbance characteristics, incorporates disturbance information directly into the optimization process, and enables coordinated operation between disturbance estimation and predictive control.

To address these challenges, a cooperative DOB-MPC architecture is proposed to mitigate the inherent bandwidth competition between disturbance estimation and predictive control. The method follows a frequency-domain decoupling principle, assigning complementary roles to the DOB and the MPC. Specifically, the DOB estimates dominant low-frequency multi-band disturbances, while the MPC compensates for time-delay-induced phase lag and performs constrained optimal control.

The disturbance observer is constructed based on a nominal model and restructured into a decoupled observer–estimator framework. By combining enhanced filtering with band-selective design, the DOB is able to extract disturbance components within the target frequency range (0.1–1 Hz) while effectively suppressing sensor noise.

The disturbance estimate is incorporated into the MPC as an additive output preview term. Instead of treating the disturbance as a constant feedforward compensation, the proposed formulation penalizes the residual output disturbance within the optimization objective. In this way, the disturbance weighting matrix $W_{d}$ directly influences the quadratic programming problem, enabling disturbance-aware control in a mathematically consistent manner.

The disturbance weighting matrix is designed as an adjustable parameter, allowing the controller to balance disturbance rejection and control effort according to the disturbance environment and estimation reliability.

Through this co-design strategy, the proposed DOB-MPC framework not only expands the effective disturbance rejection bandwidth but also mitigates phase lag induced by time delays, thereby improving overall stability and ensuring the beam-pointing accuracy required for high-quality optical communication. The effectiveness of the proposed method is validated through both simulations and experiments under various dynamic disturbance conditions.

The main contributions of this paper are summarized as follows:

- Output-disturbance-oriented modeling: The disturbance is formulated as an additive output term, which captures the physical characteristics of optical-axis perturbations in space laser communication systems.
- Frequency-domain decoupling strategy: A coordinated design of the DOB and MPC is developed in the frequency domain, where the DOB estimates dominant low-frequency disturbances and the MPC compensates for time-delay effects while enforcing constraints, thereby avoiding bandwidth competition.
- Disturbance-aware optimization formulation: The disturbance estimate is incorporated into the optimization objective through residual output penalization, enabling the disturbance weighting matrix to directly influence the quadratic programming problem.

## 2. Operational Principles and System Modeling of Coarse Tracking

### 2.1. Operational Mechanism and Multi-Frequency Disturbance Modeling

**Figure 1:**
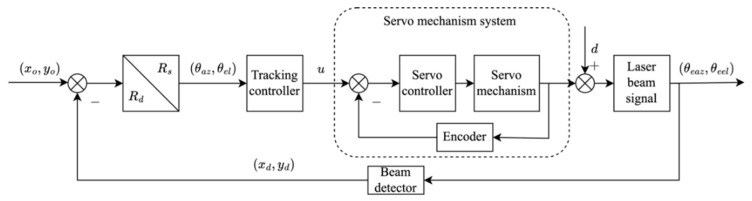
Coarse tracking systems.

Figure 1 shows the conventional coarse tracking architecture in a space laser communication system, which is used as the basis for analyzing servo-output disturbance characteristics and subsequent disturbance suppression. Spatial light detectors capture the centroid position of incident laser beams, generating position signals $\left(x_{d},y_{d}\right)$ in the detector coordinate system. The position error signal (PES) is obtained by comparing these signals with the ideal centroid position $\left(x_{o},y_{o}\right)$. These PES values are transformed from detector coordinates $R_{d}$ to mechanical coordinates $R_{s}$ via rotation matrix $T\in R^{4\times 4}$, then transmitted to the controller for real-time processing. Control algorithms compute angular correction commands executed by servo mechanisms in mechanical coordinates $\left(\theta_{az},\theta_{el}\right)$ through direct-drive DC motors. The servo mechanism closes the control loop through encoders. Output disturbance (*d*) primarily manifests at the servo system output, enabling suppression through time-domain or frequency-domain analysis of output disturbance characteristics.

The disturbance sources are first analyzed to characterize the output disturbance behavior. The disturbances (*d*) are primarily caused by the attitude and positional motions of dynamic carrier platforms, including airborne, shipborne, and satellite platforms. These disturbances can be uniformly modeled as a superposition of multi-frequency sinusoidal harmonics, as follows:

(1) $$d\left(t\right)=\sum_{k=1}^{m}A_{k}\sin\left(\omega_{k}t+\phi_{k}\right),$$

where $A_{k}$ is the disturbance amplitude, $\omega_{k}$ is the disturbance frequency, and $\phi_{k}$ is the initial phase. These disturbance parameters vary significantly across platforms. For example, satellite platforms exhibit low-amplitude (<0.05), ultra-low-frequency (<0.01 Hz) characteristics due to micro-vibration environments, while shipborne platforms show large-amplitude (>1°), low-frequency (<1 Hz) disturbances from wave excitation. A comparative summary of these platform-specific disturbance profiles is provided in Table 1.

The effective operation of laser communication on dynamic platforms therefore requires addressing dominant, low-frequency, and high-amplitude attitude motions, as they can otherwise lead to severe link degradation. To enhance the environmental adaptability of control algorithms, this study specifically focuses on high-speed maneuvering platform scenarios, where the disturbance components of interest satisfy:

(2) $$A_{k}>1{}^{\circ},\omega_{k}>0.1\;Hz.$$

The shipboard or aircraft environment imposes the most demanding requirements on a control system, particularly in terms of dynamic range and tracking bandwidth. This challenge is compounded by time delays introduced when observing external disturbances through spot detectors (typically CMOS cameras). Integration times ranging from 1 to 10 ms lead to equivalent feedback delays, which reduce system response and disturbance rejection. Generally, expanding system bandwidth can enhance dynamic response, but this simultaneously amplifies high-frequency noise. The time delays further erode phase margins, preventing effective broadband disturbance rejection and degrading stability. Under multi-frequency disturbances, the detrimental effects of time delays become more pronounced, making it particularly complex to maintain both sufficient disturbance rejection bandwidth and system stability.

### 2.2. Motion Modeling and Problem Analysis

For controller design and analysis, the gimbal servo mechanism is abstracted as a DC motor-driven system. A second-order state-space model is formulated with the mechanical position θ(t) and angular velocity ω(t) as state variables, defined as $X\left(t\right)={\left[\theta \left(t\right),\omega \left(t\right)\right]}^{T}$. The control input is the armature current U(t) = ia(t). While the external physical disturbance initially enters the system as a load torque $T_{L}\left(t\right)$ at the input, its cumulative effect is mathematically mapped as an equivalent lumped kinematic disturbance d(t) at the system output for the subsequent observer design.

The continuous-time state-space representation of the system can be given by:

(3) $$\frac{d}{dt}\left[\begin{array}{c} \theta \left(t\right)\\ \omega \left(t\right) \end{array}\right]=\underset{\mathrm{Ax}}{\underbrace{\left[\begin{array}{cc} 0 & 1\\ 0 & -\frac{B}{J} \end{array}\right]}}X\left(t\right)+\underset{\mathrm{Bu}}{\underbrace{\left[\begin{array}{c} 0\\ \frac{K_{t}}{J} \end{array}\right]}}u\left(t\right)+\underset{\mathrm{Ed}}{\underbrace{\left[\begin{array}{c} 0\\ -\frac{1}{J} \end{array}\right]}}d\left(t\right),$$

with full-state measurement output:

(4) $$y\left(t\right)=\left[\begin{array}{cc} 1 & 0\\ 0 & 1 \end{array}\right]x\left(t\right),$$

where *J* is the moment of inertia, *B* is the viscous friction coefficient, and *K_t_* is the motor torque constant.

**Figure 2:**
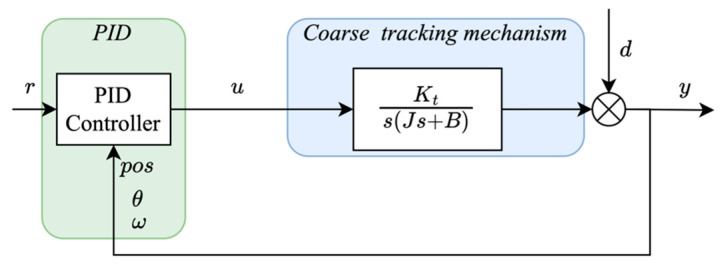
Traditional PID tracking systems.

Equations (3) and (4) constitute the dynamic model of the motor, and a cascaded PID control architecture is commonly adopted to drive the coarse tracking systems, as shown in Figure 2. Although structurally simple, this approach exhibits several critical limitations when subjected to multi-frequency disturbances and system delays. Firstly, the strong coupling between the inner velocity loop and the outer position loop greatly increases the difficulty of parameter tuning. Moreover, while PI controllers are commonly used in practice to suppress sensor noise, they are inherently inadequate for rejecting non-constant disturbances (e.g., sinusoidal components) without introducing steady-state error. Furthermore, system delays constrain the permissible controller gains needed to achieve faster response, thereby fundamentally limiting the system’s capability to suppress multi-frequency disturbances. As the disturbance spectrum becomes richer, the cumulative effect of residual errors across frequency bands leads to pronounced tracking deviations, potentially exceeding the fine tracker’s field of view [25]. This analysis emphasizes the inherent insufficiency of traditional PID-based control schemes and the critical necessity for an advanced control framework capable of concurrently addressing multi-frequency disturbance estimation and time-delay compensation.

## 3. Control System Design

A novel control architecture integrating a multi-frequency DOB with MPC is proposed to improve dynamic response and residual error suppression under multi-frequency disturbances. This co-design leverages the precise disturbance estimation capability of the DOB and the inherent time-delay compensation and optimization prowess of MPC. By incorporating the disturbance estimates into the MPC’s predictive optimization process, the proposed architecture achieves effective multi-frequency disturbance suppression, enhancing both the dynamic response and steady-state tracking accuracy of the system significantly.

### 3.1. Observer Design

**Figure 3:**
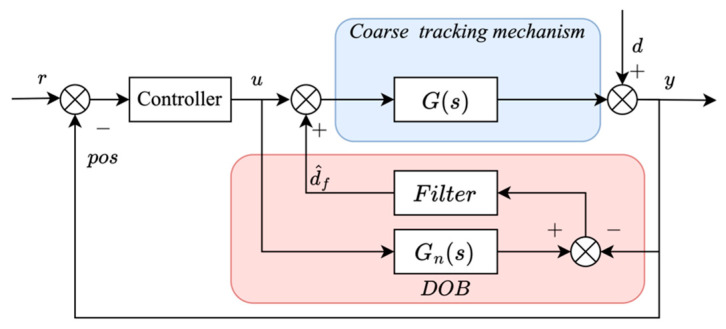
Improved multi-frequency output disturbance DOB.

An improved DOB architecture for suppressing output-type multi-frequency disturbances is proposed, as shown in Figure 3. The estimation error signal is obtained from the discrepancy between the measured plant output and the nominal output predicted by the disturbance-free plant model. This discrepancy represents the lumped output disturbance caused by optical-axis perturbations and platform-induced angular motions. The error signal is then processed through a low-pass filter to reconstruct the dominant disturbance components while reducing measurement noise, thereby improving the robustness and reliability of the DOB system.

Based on the nominal model from Equation (3), the nominal plant model can be described by:

(5) $$\dot{x}\left(t\right)=Ax\left(t\right)+Bu\left(t\right)\;y\left(t\right)=Cx\left(t\right)+d\left(t\right),$$

where $d\left(t\right)=\sum_{k=1}^{m}A_{k}\sin\left(\omega_{k}t+\phi_{k}\right)$ represents multi-frequency output disturbance caused by environmental disturbances. Unlike traditional observers targeting input disturbances, this paper directly constructs a state observer based on the nominal model:

(6) $$\dot{\hat{x}}\left(t\right)=A\hat{x}\left(t\right)+Bu\left(t\right)+L\left(y\left(t\right)-C\hat{x}\left(t\right)\right),$$

where the gain matrix L is designed such that (A−LC) is Hurwitz, ensuring the state estimation error $\left(e_{x}=x-\hat{x}\right)$ exponentially converges. The output disturbance estimation can then be obtained as follows:

(7) $$\hat{d_{o}}\left(t\right)=y\left(t\right)-C\hat{x}\left(t\right)=d\left(t\right)+Ce_{x}\left(t\right).$$

To separate multi-frequency disturbances and suppress sensor drift and high-frequency noise, a composite filter is designed. To extract the low-frequency components of the target disturbance, a digital notch filter is introduced:

(8) $$N\left(z\right)=\frac{1-2\cos\left(\omega_{drift}T\right)z^{-1}+z^{-2}}{1-2\rho \cos\left(\omega_{drift}T\right)z^{-1}+\rho^{2}z^{-2}},$$

where $\omega_{\mathrm{drift}}$ is drift frequency and $\rho \in \left(0,1\right)$ controls stopband width. Combined with low-pass filter *Q(s)*, the final multi-frequency disturbance estimation can be expressed as:

(9) $$\hat{d_{f}}\left(s\right)=\underset{\mathrm{Low}\text{-}\mathrm{frequency}\;\mathrm{enhancement}}{\underbrace{Q\left(s\right)}}\cdot \underset{\mathrm{Drift}\;\mathrm{suppression}}{\underbrace{N\left(s\right)}}\cdot \hat{d}\left(s\right).$$

This architecture extracts target band disturbances through *Q(s)*, while *N(s)* forms stopbands with depth no less than 40 dB below 0.01 Hz and above 10 Hz, effectively eliminating sensor drift effects on estimation accuracy. Stability analysis shows that when $|W\left(s\right)\left(1-Q\left(s\right)N\left(s\right)\right){|}_{\infty }<1$, the closed-loop system remains robustly stable under model parameter perturbations, where *W(s)* is a weighting function reflecting unmodeled dynamics. The core advantage of this architecture is its effective observation of coarse tracking system output disturbances, solving the multi-frequency disturbance feedback problem and providing key input for subsequent reduction in system tracking residuals.

### 3.2. Model Predictive Controller Design

**Figure 4:**
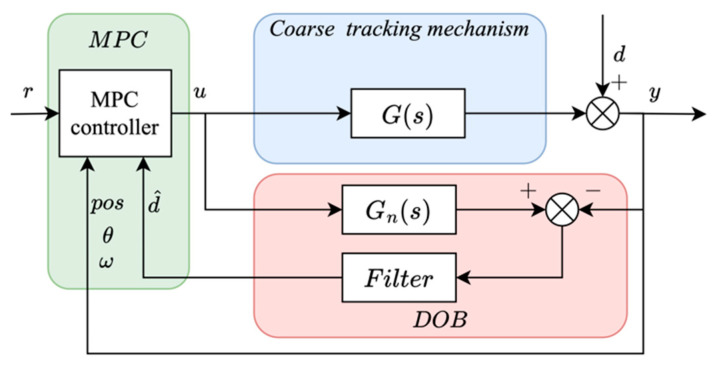
DOB-MPC control structure.

Conventional MPC frameworks mainly improve tracking accuracy by optimizing the control input sequence, but they have limited capability in suppressing persistent multi-frequency output disturbances. In the considered space laser communication system, the dominant disturbance originates from optical-axis perturbations and platform motion and therefore acts directly on the tracking output rather than through the plant input channel. To address this issue, the disturbance estimate $\hat{d}$ provided by the DOB is introduced into the MPC prediction as an additive output preview term. This enables the predictive controller to generate a compensating output response in advance and reduce the residual output disturbance. The overall cooperative control structure is illustrated in Figure 4.

In the considered coarse tracking system, the dominant disturbance originates from optical-axis perturbations and acts directly on the output, as shown in Figure 4. Therefore, the disturbance is modeled as an additive output disturbance rather than an input-side uncertainty.

The predicted output over the horizon can be expressed as:

(10) $$y\left(k+i|k\right)=CA_{d}^{i}x\left(k\right)+\sum_{j=0}^{i-1}CA_{d}^{i-j-1}B_{d}u\left(k+j|k\right)+\hat{d}\left(k\right),$$

where the disturbance estimate $\hat{d}\left(k\right)$ provided by the DOB is assumed to remain constant over the prediction horizon. Accordingly, the predicted output vector is:

(11) $$Y=\Psi x\left(k\right)+\Theta U+D,$$

where D represents the predicted output disturbance sequence constructed from the DOB estimate:

(12) $$\Psi =\left[CA;CA^{2};\ldots ;CA^{N_{p}}\right],$$

and Θ denotes the Toeplitz matrix:

(13) $$\Theta =\left[\begin{array}{cccc} CB & 0 & \ldots & 0\\ CAB & CB & \ldots & 0\\ \vdots & \vdots & \ddots & \vdots \\ A^{N_{p}-1}B & CA^{N_{p}-2}B & \ldots & {CA}^{N_{p}-N_{c}}BC \end{array}\right],$$

(14) $$\Phi =I_{N_{p}},D={\left[\hat{d}\left(k|k\right);\hat{d}\left(k+1|k\right);\ldots ;\hat{d}\left(k+N_{p}|k\right)\right]}^{T},$$

In this work, $\hat{d}\left(k+i|k\right)$ is assumed to be constant, i.e., $\hat{d}\left(k+i|k\right)=\hat{d}\left(k\right)$.

A quadratic cost function incorporating the disturbance estimate is designed:

(15) $$J={∥Y_{ref}-\left(Y_{f}+\Theta U\right)∥}_{Q}^{2}+{∥\Theta U+D∥}_{W_{d}}^{2}+{∥U∥}_{R}^{2},$$

where $Y_{f}=\Psi x\left(k\right)$ denotes the free response generated by the current state, ΘU denotes the output response induced by the future control sequence, and *D* denotes the predicted additive output disturbance sequence provided by the DOB.

It should be emphasized that **D** is treated as a known disturbance preview over the prediction horizon and is not an optimization variable. Therefore, the weighting matrix $W_{d}$ does not penalize the disturbance estimate itself. Instead, it penalizes the control-dependent residual output disturbance after compensation, defined as,

(16) $$e_{d}(U)=\Theta U+D.$$

This formulation encourages the predictive controller to generate an output response ΘU that counteracts the predicted disturbance **D**. Since $e_{d}(U)$ explicitly depends on the decision variable U, the disturbance weighting matrix $W_{d}$ directly affects the resulting quadratic programming problem.

The constraints considered in the QP include input amplitude limits, input increment limits, and a terminal set constraint imposed on the nominal predicted state:

(17) $$x{\left(k+N_{p}|k\right)}^{T}Px\left(k+N_{p}|k\right)\leq \gamma ,$$

where *P* is obtained by solving the Riccati equation:

(18) $$A_{d}^{T}PA_{d}-P-A_{d}^{T}PB_{d}{\left(R+B_{d}^{T}PB_{d}\right)}^{-1}B_{d}^{T}PA_{d}+Q_{x}=0,$$

where $Q_{x}⪰0$ is the state weighting matrix used for the terminal cost. After expanding Equation (15) and omitting the terms independent of U, the optimization problem is transformed into the following QP:

(19) $$\underset{U}{\min}J=U^{T}HU+2f^{T}U,\;subject\;to:u_{min}\leq u\left(k+i\right)\leq u_{max},\;\Delta u_{min}\leq \Delta u\left(k+i\right)\leq {\Delta u}_{max},\;\;x{\left(k+N_{p}|k\right)}^{T}Px\left(k+N_{p}|k\right)\leq \gamma ,$$

where $i=0,\ldots ,N_{c}-1,$ and $H=\Theta^{T}Q\Theta +R+{\Theta^{T}W}_{d}\Theta$ and $f={{\Theta^{T}W}_{d}D-\Theta }^{T}Q\left(Y_{ref}-Y_{f}\right)$. Here, Q⪰0, $W_{d}⪰0$, and R≻0. Therefore, H is positive definite, which ensures that the QP is convex. This formulation is consistent with the control structure shown in Figure 4, where the disturbance acts directly on the system output. The control objective is therefore to generate an output response that compensates for the disturbance effect.

Unlike formulations that directly penalize the fixed disturbance preview **D**, the proposed formulation penalizes the control-dependent residual disturbance ΘU+D. Therefore, the weighting matrix $W_{d}$ directly influences the quadratic program through both the Hessian and gradient terms, thereby ensuring mathematical consistency.

The optimal control input sequence over the prediction horizon is derived by solving the optimization problem. Following the receding horizon principle, only the first element of this sequence is implemented at each time step:

(20) $$u\left(k\right)=u^{*}\left(k|k\right).$$

To analyze the closed-loop stability, the disturbance is modeled as an additive output perturbation:

(21) $$y_{k}=Cx_{k}+d_{k}.$$

Let the disturbance estimation error be defined as:

(22) $$\tilde{d_{k}}=d_{k}-\hat{d_{k}},\;\;||\tilde{d_{k}}||\leq \epsilon .$$

The tracking error can be decomposed as:

(23) $$e_{k}=y_{k}-r_{k}=\bar{e_{k}}+\tilde{d_{k}},$$

where $\bar{e_{k}}=Cx_{k}+\hat{d_{k}}-r_{k}$ denotes the compensated tracking error associated with the disturbance estimate used by the MPC.

Under standard MPC assumptions and with the disturbance preview fixed over one sampling step, the optimal cost $V_{N}\left(x_{k},\hat{D_{k}}\right)$ can be used as a Lyapunov-like function for the compensated prediction model and satisfies the following inequality:

(24) $$V_{N}\left(x_{k+1},\hat{D_{k}}\right)-V_{N}\left(x_{k},\hat{D_{k}}\right)\leq -\alpha ||\bar{e_{k}}{\left|\right|}^{2}+\beta \epsilon^{2}.$$

Therefore, the compensated tracking error $\bar{e_{k}}$ is uniformly ultimately bounded (UUB), and satisfies:

(25) $${\mathrm{lim sup}}_{k\to \infty }\left|\right|\bar{e_{k}}||\leq \sqrt{\frac{\beta }{\alpha }}\epsilon .$$

Since $e_{k}=\bar{e_{k}}+\tilde{d_{k}}$ and $||\tilde{d_{k}}||\leq \varepsilon$, the actual tracking error is also UUB and satisfies:

(26) $${\mathrm{lim sup}}_{k\to \infty }\left|\right|e_{k}||\leq (1+\sqrt{\frac{\beta }{\alpha }})\epsilon .$$

This expression indicates that the achievable tracking accuracy is primarily limited by the disturbance estimation error. The constants α and β are related to the MPC weighting matrices and the closed-loop prediction model. It should be noted that $W_{d}$ does not directly reduce the disturbance estimation error ε. Instead, it reshapes the MPC optimization through the residual-disturbance penalty $||\Theta U+D{\left|\right|}_{W_{d}}^{2}$, thereby affecting the compensated tracking error and reflecting the trade-off among disturbance rejection, reference tracking, and control effort.

The proposed DOB-MPC framework incorporates disturbance estimation into predictive optimization, enabling enhanced suppression of persistent multi-frequency disturbances. Its effectiveness is further validated through simulations and experiments in the following section.

## 4. Simulation and Experimental Validation

### 4.1. Simulation Analysis

**Figure 5:**
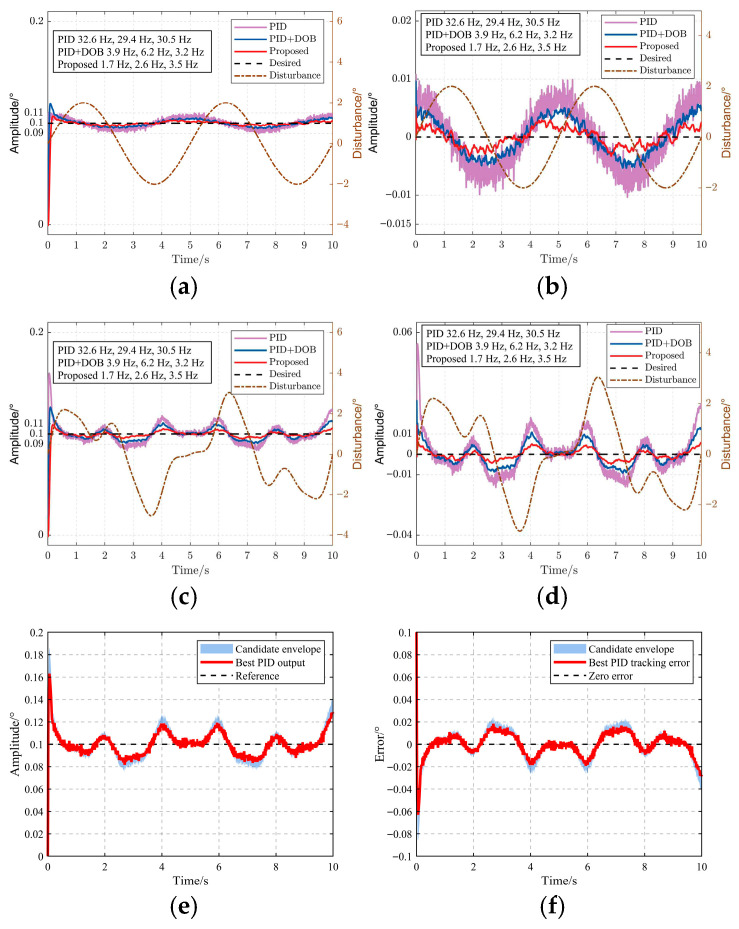
Tracking performance under external angular disturbances. (**a**) Step response under a 0.2 Hz, 1° sinusoidal disturbance. (**b**) Corresponding tracking error for the case in (**a**). (**c**) Step response under multi-frequency sinusoidal disturbance. (**d**) Corresponding tracking error for the case in (**c**). (**e**) PID tuning response envelope. (**f**) PID tuning error envelope.

To evaluate the residual disturbance suppression capability of the proposed control system, simulations were conducted using the system dynamics defined by Equations (3) and (4). The evaluation considers both step tracking and disturbance rejection under single-frequency and multi-frequency angular disturbances, as shown in Figure 5.

As shown in Figure 5a,c, the conventional PID controller exhibits a faster rise response but suffers from large overshoot and noticeable oscillations. This behavior is mainly caused by the high feedback gains required to suppress external disturbances in the presence of a 10 ms system delay. Although higher gains improve the initial response speed, they also reduce damping and increase residual oscillations. The PID parameters used in the comparison were tuned to achieve the best compromise between response speed, overshoot, and steady-state tracking error; further increasing the integral or derivative gains was found to either amplify oscillations or degrade disturbance rejection.

The introduction of a disturbance observer improves the disturbance attenuation capability of PID control. As a result, PID-DOB significantly reduces the residual tracking error compared with conventional PID. However, since PID-DOB still relies on a feedback-dominated compensation mechanism, its ability to handle time-delay-induced phase lag and multi-frequency disturbance coupling remains limited.

In contrast, the proposed DOB-MPC method achieves a more damped and smoother response. Although its rise time is slightly longer than that of PID and PID-DOB, the overshoot and residual oscillation are substantially reduced. This indicates that the proposed method does not improve performance by increasing feedback aggressiveness but by using disturbance prediction and constrained optimization to generate a smoother control trajectory. Therefore, the slower rise time represents a deliberate trade-off for improved damping, reduced overshoot, and better steady-state tracking accuracy.

The corresponding tracking errors in Figure 5b,d further confirm this behavior. Under both single-frequency and multi-frequency disturbances, the PID controller produces tracking errors exceeding the allowable 0.01° stability margin. PID-DOB reduces the error amplitude by compensating for dominant disturbance components, but residual errors remain due to limited predictive capability. The proposed DOB-MPC method further suppresses the tracking error and maintains it close to or below the 0.01° threshold, which is required to keep the beam within the field of view of the fine tracking subsystem. To ensure a fair comparison, the PID parameters were tuned over a broad candidate range. As shown in Figure 5e,f, the selected PID output lies within the candidate envelope and provides a near-optimal compromise between response speed, overshoot, and residual tracking error. A further gain increase was found to amplify oscillations under multi-frequency disturbances. Thus, the PID baseline used for comparison represents the best practical tuning result under the considered conditions.

The quantitative results in Table 2 further confirm this behavior. Across the four multi-frequency disturbance conditions, the average overshoot is reduced from 40.69% for PID and 23.55% for PID-DOB to 8.05% for DOB-MPC, corresponding to reductions of approximately 80.2% and 65.8%, respectively. Similarly, the average peak-to-peak tracking error decreases from 0.0313° for PID and 0.0178° for PID-DOB to 0.0083° for DOB-MPC, representing reductions of approximately 73.5% and 53.4%, respectively.

Overall, the simulation results demonstrate that DOB-MPC improves disturbance rejection not by accelerating the initial step response, but by reducing overshoot, suppressing residual oscillations, and maintaining stable tracking accuracy under multi-frequency disturbances.

The relationship between tracking error and optical communication performance can be described by the coupling efficiency model, which decreases exponentially with pointing error. Specifically, the coupling efficiency can be approximated as

$$\eta =\exp\left(-\frac{\theta^{2}}{2\sigma^{2}}\right),$$

where θ is the pointing error and σ represents the beam divergence parameter. This relationship indicates that reducing residual pointing error is expected to improve optical power coupling, which can further support a higher received signal-to-noise ratio (SNR) and a lower bit error rate (BER). However, it should be noted that the present experiments focus on mechanical tracking performance, and direct measurements of received optical power, coupling efficiency, SNR, and BER are not included. Therefore, the communication-link improvement is discussed as a model-based implication rather than a direct experimental verification.

### 4.2. Experimental Validation

**Figure 6:**
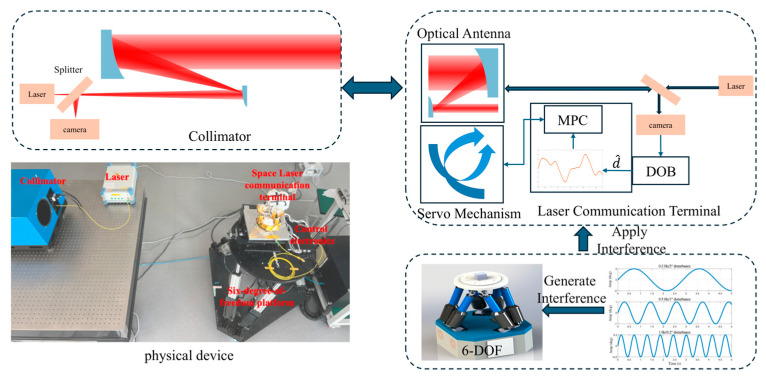
Disturbance emulation and suppression experimental platform.

An experimental platform was established to evaluate the proposed algorithm under simulated space disturbance conditions, as shown in Figure 6. The transmitting side employs a custom large-aperture collimator (150 mm, 30 μrad divergence) to generate a quasi-parallel Gaussian beam, emulating long-distance optical links. The receiving terminal, with a 60 mm aperture and an 800 mm focal length optical antenna, is equipped with a tracking camera (5 μm pixel size, corresponding to an angular resolution of approximately 6.25 μrad/pixel). The terminal is mounted on a six-degree-of-freedom motion platform that generates angular disturbances in the range of 0.01–10 Hz with amplitudes up to several degrees, which would drive the beam out of the camera field of view (~6 mrad) without compensation. In closed-loop operation, the servo system suppresses these disturbances, and the camera captures only the residual pointing error within a small angular range. A dual-function collimator with a beam splitter provides both the reference beam and error detection: the detected spot position is used to compute tracking errors, which are processed by the disturbance observer (DOB) and fed into the model predictive controller (MPC) for iterative compensation.

**Figure 7:**
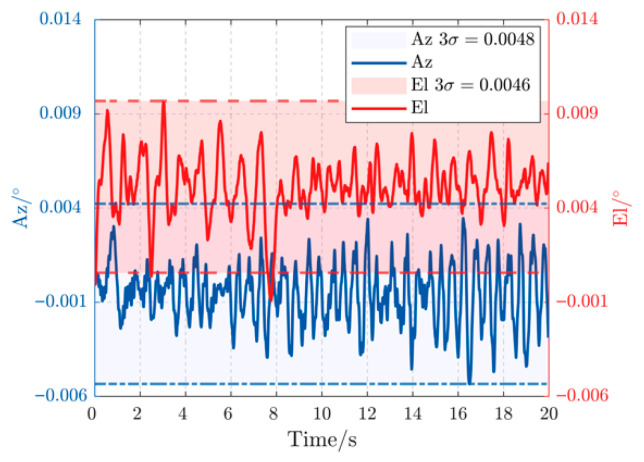
Error curves under 0.2 Hz, 3° disturbance.

**Figure 8:**
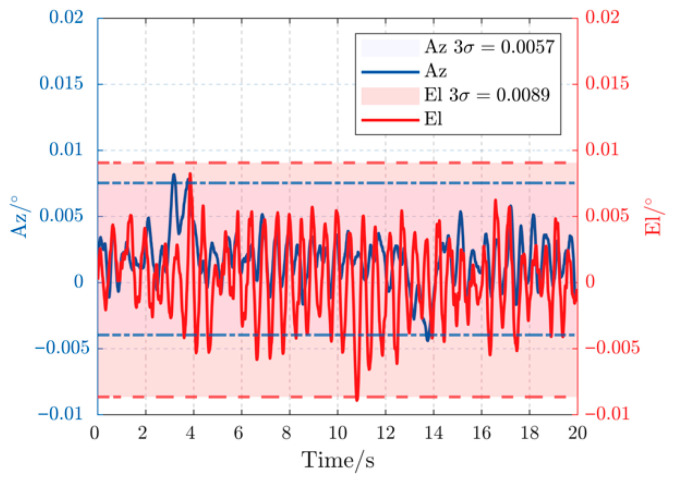
Error curves under 0.5 Hz, 3° disturbance.

Figure 7 displays the experimental tracking error for both azimuth (Az) and elevation (El) axes under a 0.2 Hz, 3° disturbance. The proposed controller confines the peak-to-peak error to less than 0.006°, demonstrating exceptional rejection of low-frequency, high-amplitude perturbations. The error curves are smooth and symmetric, indicating that the proposed DOB is accurately estimating and compensating the disturbance without introducing high-frequency noise. When the disturbance frequency is increased to 0.5 Hz in Figure 8, the peak-to-peak error gracefully degrades to below 0.009°. This slight performance reduction is expected as the disturbance bandwidth approaches the system’s control bandwidth, yet the controller maintains robust stability and precision, comfortably within the sub-milliradian requirement.

**Figure 9:**
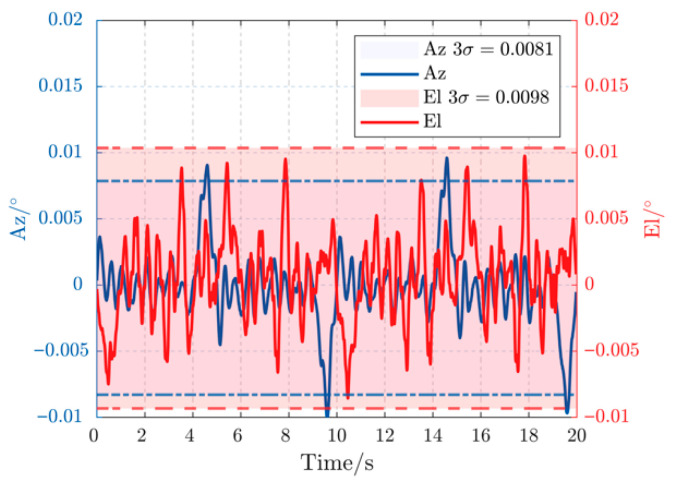
Multi-frequency disturbance error curves.

The ultimate test of the proposed architecture is its performance under spectrally rich disturbances. Figure 9 shows the system’s response to a composite disturbance (0.2 Hz/2° + 0.5 Hz/1° + 1 Hz/0.2°). Remarkably, the tracking accuracy does not exhibit significant degradation compared to the single-frequency cases. The error remains bounded within 0.01°, proving that the DOB’s composite filter successfully decomposes the complex disturbance into its constituent frequencies, and the MPC effectively integrates this composite estimate into its optimization, handling multiple disturbance bands concurrently.

**Figure 10:**
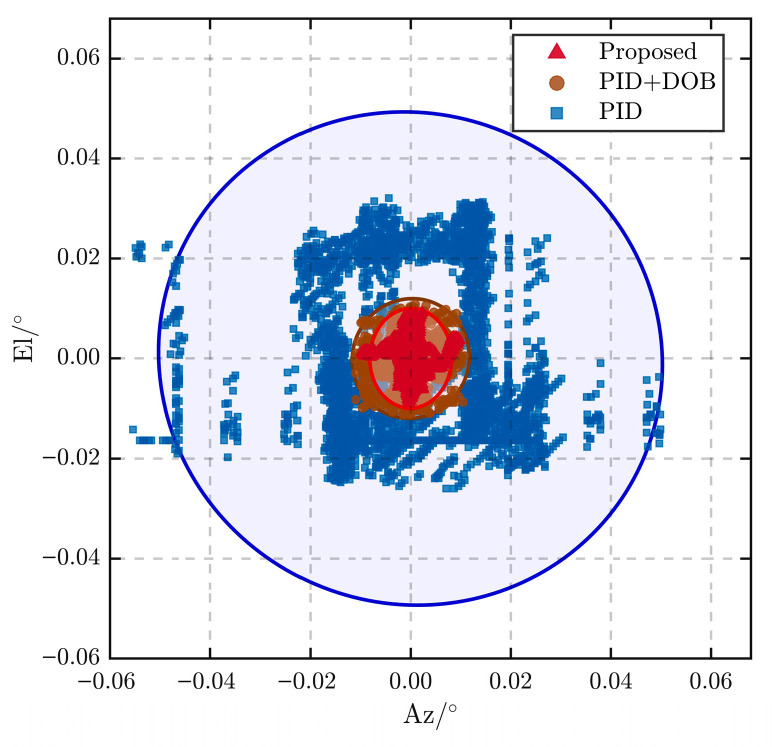
Tracking error comparison among PID, PID-DOB, and DOB-MPC under multi-frequency disturbances.

A direct quantitative comparison among the conventional PID, PID + DOB, and the proposed DOB-MPC methods under multi-frequency disturbances is shown in Figure 10. The conventional PID exhibits a peak-to-peak tracking error of approximately 0.055° with a standard deviation of 0.0165°, indicating significant fluctuation. The PID + DOB method reduces these to 0.022° and 0.0039°, respectively, demonstrating effective disturbance attenuation. In comparison, the proposed DOB-MPC further improves performance, achieving a peak-to-peak error of 0.0098° and a standard deviation of 0.0033°. More importantly, by integrating disturbance estimation with predictive optimization, the proposed method not only reduces error magnitude but also enhances temporal consistency, as reflected by the tighter clustering of tracking points in Figure 10. This ensures that the tracking error remains within 0.01°, satisfying the requirement that coarse tracking stays within the field of view of the fine tracking subsystem.

The proposed DOB-MPC architecture was systematically validated through both simulations and experiments. Compared with conventional PID and PID + DOB controllers, the proposed method demonstrates superior performance in suppressing multi-frequency disturbances, reducing tracking error magnitude, and improving stability.

Experimental results on a 6-DOF motion platform show that, while PID + DOB effectively reduces disturbance amplitude, the proposed approach achieves further performance gains by incorporating predictive optimization. This enables simultaneous suppression of residual fluctuations and enhancement of tracking consistency under complex disturbance conditions.

The above results confirm that the integration of frequency-decoupled disturbance estimation with model predictive control provides a practical and effective solution for high-precision coarse tracking systems, meeting the stringent requirements of space laser communication applications.

## 5. Conclusions

In this paper, the issues of unsatisfactory dynamic response and residual error suppression for coarse tracking systems under multi-frequency disturbances are addressed through the design of a cooperative DOB-MPC architecture. A frequency-domain decoupling scheme is adopted to achieve separate regulation tasks. Disturbance estimation for multi-frequency components is completed via DOB, while MPC is configured to mitigate time delay and fuse disturbance information into receding-horizon optimization. Simulation and experimental results validate that, under challenging composite disturbances, the proposed method achieved an 82.2% reduction in peak-to-peak tracking error compared to conventional PID control and a further 21.4% improvement over the PID-DOB method. Crucially, by maintaining the tracking precision below the 0.01° threshold, the controller not only satisfies the mechanical requirements of the fine tracker but also helps improve optical coupling efficiency at the receiver. This sub-milliradian stabilization translates directly into improved optical link performance, providing the high signal-to-noise ratio and low bit error rate essential for reliable spatial data transmission, thereby validating the engineering applicability of the DOB-MPC architecture for robust space laser communication systems.

## Figures and Tables

**Table 1:** Platform-specific attitude disturbance profiles.

Platform	Primary Disturbance Source	Frequency Range	Typical Angular Amplitude
Spaceborne	Attitude Maneuvers and Drift	<0.1 Hz	0.05–0.5°
Airborne	Aeroelastic and Piloting Motions	0.1–5 Hz	0.3–1.5°
Shipborne	Sea-Induced Sway (Roll/Pitch)	0.05–1 Hz	1–5° (and beyond)

**Table 2:** Quantitative performance comparison under multi-frequency disturbance conditions.

Conditions		0.2 Hz + 0.5 Hz	0.2 Hz + 1 Hz	0.5 Hz + 1 Hz	0.2 Hz + 0.5 Hz + 1 Hz
Overshoot (%)	PID	40.071	18.336	24.575	59.796
	PID-DOB	24.596	21.147	22.033	26.413
	DOB-MPC	8.700	7.128	7.282	9.101
Risetime (s)	PID	0.012	0.014	0.014	0.011
	PID-DOB	0.034	0.036	0.035	0.033
	DOB-MPC	0.097	0.071	0.070	0.097
Peak-to-peak error (°)	PID	0.035	0.026	0.023	0.041
	PID-DOB	0.020	0.015	0.014	0.022
	DOB-MPC	0.009	0.0071	0.007	0.010
RMS error (°)	PID	0.009	0.006	0.007	0.010
	PID-DOB	0.007	0.006	0.006	0.006
	DOB-MPC	0.007	0.007	0.007	0.007

Note: Disturbances at 0.2 Hz have an amplitude of 2°, at 0.5 Hz of 1°, and at 1 Hz of 0.2°.

## Data Availability

The data presented in this study are available on request from the corresponding author.

## Abbreviations

The following abbreviations are used in this manuscript:

MPC	model predictive control
DOB	disturbance observer
PAT	pointing, acquisition, and tracking
